# Immune Maturation Effects on Viral Neutralization and Avidity of Hyperimmunized Equine Anti-SARS-CoV-2 Sera

**DOI:** 10.3390/antib11010003

**Published:** 2022-01-02

**Authors:** Myriam Belén González Viacava, Augusto Varese, Ignacio Mazzitelli, Laura Lanari, Lucía Ávila, María Julia García Vampa, Jorge Geffner, Osvaldo Cascone, José Christian Dokmetjian, Adolfo Rafael de Roodt, Matías Fingermann

**Affiliations:** 1 Instituto Nacional de Producción de Biológicos (INPB), ANLIS “Dr. Carlos G. Malbrán”, Vélez Sársfield 563, Buenos Aires 1282, Argentina; belengviacava@gmail.com (M.B.G.V.); llanari@anlis.gob.ar (L.L.); lavila@anlis.gov.ar (L.Á.); juli.vampa@gmail.com (M.J.G.V.); ocascone@hotmail.com (O.C.); jdokmetjian@anlis.gob.ar (J.C.D.); aderoodt@anlis.gob.ar (A.R.d.R.); 2 Instituto de Investigaciones Biomédicas en Retrovirus y SIDA (INBIRS), Universidad de Buenos Aires (UBA) and Consejo Nacional de Investigaciones Científicas y Técnicas (CONICET), Paraguay 2155, 11th Floor, Buenos Aires 1113, Argentina; augustovarese89@hotmail.com (A.V.); mazzitelli.ignacio@gmail.com (I.M.); jorgegeffner@gmail.com (J.G.); 3 Consejo Nacional de Investigaciones Científicas y Técnicas (CONICET), Godoy Cruz 2290, Buenos Aires 1425, Argentina; 4 Instituto de Nanobiotecnología (NANOBIOTEC), Universidad de Buenos Aires (UBA) and Consejo Nacional de Investigaciones Científicas y Técnicas (CONICET), Junín 956, Buenos Aires 1113, Argentina; 5 Cátedra de Toxicología, Facultad de Medicina, Universidad de Buenos Aires, Paraguay 2155, Buenos Aires 1113, Argentina

**Keywords:** COVID-19, anti-SARS-CoV-2, hyperimmunization, therapeutics, immune maturation, hyperimmune equine

## Abstract

Mass-vaccination against COVID-19 is still a distant goal for most low-to-middle income countries. The experience gained through decades producing polyclonal immunotherapeutics (such as antivenoms) in many of those countries is being redirected to develop similar products able to neutralize SARS-CoV-2 infection. In this study we analyzed the biological activity (viral neutralization or NtAb) and immunochemical properties of hyperimmune horses’ sera (HHS) obtained during initial immunization (I) and posterior re-immunization (R) cycles using the RBD domain of the SARS-CoV-2 spike protein as antigen. HHS at the end of the R cycle showed higher NtAb titers when compared to those after the I cycle (35,585 vs. 7000 mean NtAb, respectively). Moreover, this increase paralleled an increase in avidity (95.2% to 65.2% mean avidity units, respectively). The results presented herein are relevant for manufacturers of these therapeutic tools against COVID-19.

## 1. Introduction

Since the end of 2019, healthcare systems all around the world are highly stressed due to the SARS-CoV-2 pandemic. Despite the unprecedented pace at which new vaccines and therapeutics are being tested, licensed, and introduced against a novel disease such as COVID-19, the struggle to control this disease at global scale is still far from over [1]. Many effective and safe vaccines are currently licensed and mass vaccination has started in several countries. Unfortunately, due to the inequity in distribution and under-optimal production rate, most low-to-middle income countries are suffering shortages on vaccine availability to complete their immunization plans. Viral spread in those countries continues to increase, further stressing their healthcare systems and intensive care units, thus favoring conditions for the appearance of SARS-CoV-2 variants [2]. There is a growing concern about one of these variants, designated Delta, for which current available vaccines are less effective against infection and household spread [3]. Thus, until vaccine availability for mass vaccination becomes a reality around the world, combined to the emergence of highly infective variants, it could be stated that therapeutic agents will continue at the center of the fight against this disease. From the many treatments and therapeutics under evaluation, few have reached approval for their use or, in some cases, emergency use authorization from recognized regulatory agencies. These include the drug remdesivir, monoclonal antibodies (bamlanivimab and casirivimab-imdevimab cocktail), convalescent plasma and, recently, equine polyclonal F(ab’)_2_ antibody fragments (EpAbs) [4,5,6,7,8]. EpAbs’s production is based on the same technology used for antivenoms production. Decades of experience on the production of antivenoms and already established safety profiles of these biotherapeutics, make EpAbs a potential emergency aid to help fight COVID-19 on the verge of a sanitary crisis [9].

Hyperimmunized horses’ plasma (HHP) is the starting material for EpAbs production. During HHP production, horses are hyperimmunized with inactivated SARS-CoV-2 virions or selected viral subunits in order to achieve high titers of neutralizing circulating antibodies [10,11,12,13,14]. The Spike protein of Coronaviruses, a complex homotrimeric membrane-protein, is central to host-cell attachment and invasion [15]. Each monomer consists of two subunits, the S1 subunit, comprising the receptor binding regions, and the S2 subunit, harboring the membrane-fusion and cell-invasion machinery. The Receptor Binding Domain (RBD), located in the S1 subunit of SARS-CoV-2 Spike protein, is critical for binding to the main host-cell receptor protein: angiotensin-converting enzyme 2 (ACE2) [16]. Thus, most HHP studies have focused on the Spike protein for antigen preparation: a prefusion conformation stabilized version of the Spike protein ectodomain [10], its RBD domain [11,14] or the S1 subunit alone or combined to the Nucleoprotin and Spike E-M mosaic proteins [12]. All reports on anti-SARS-CoV-2 HHP preparation analyze and describe initial hyperimmunization cycles on reduced groups of no more than five production animals. However, when large animals such as horses are destined to HHP production, they are submitted to tens of hyperimmunization cycles with the same antigen throughout their lives. Antibody class-switching, immunological memory induction and affinity maturation are well known processes that take place in vertebrates as consequence of repeated cycles of immunization [17]. Surprisingly, the effect of these cycles on circulating antibodies quality has attracted scarce attention in literature. In this study we compared relevant quantitative and qualitative aspects during the initial (I) and the following re-hyperimmunization (R) cycles on 24 horses’ sera destined to an EpAbs candidate production currently under clinical evaluation (Clinicaltrial.gov (accessed on 14 July 2021) identifier NCT04913779). We hope the results presented herein help other EpAbs producers to improve their antivirals production during the current struggle against the SARS-CoV-2 pandemic.

## 2. Materials and Methods

Antigen Production: rRBD was produced and provided by the COVIDAR Group. Basically, a plasmid harboring the RBD sequence (codons 319–541) of a mammalian cell codon-optimized nucleotide sequence version of the Spike protein sequence (GenBank: MN908947.3) from the first virus isolate, Wuhan-Hu-1, of SARS-CoV-2 along with the signal peptide (codons 1–14) plus an hexahistidine tag was kindly gifted by Florian Krammer [18]. The recombinant sequence of interest was then subcloned to pCDNA human expression vector and used for transient expression of a recombinant version of the RBD protein (rRBD) after transfection in FreeStyle 293-F (293-F) cells, as described by Ojeda et al. [19]. Transfected cells were cultured for five days in Expi293 expression medium at 33 °C under 8% CO_2_ atmosphere, harvested by centrifugation and rRBD was purified from culture supernatant by affinity chromatography (HisTrap excel columns, Cytiva, Marlborough, MA, USA). After buffer exchange (NAP-5 columns, Cytiva, Marlborough, MA, USA) typical yields of 45 rRBD mg/L and 98% purity were observed. A representative SDS-PAGE characterization of a rRBD batch is shown in Figure S1.

Horse immunization: The sera from 24 mixed-breed 4–10 years-old, 300–450 kg horses from current INPB’s anti-SARS-CoV-2 production stud were analyzed in this study. During the I cycle horses were initially primed subcutaneously with 0.5 mg of rRBD in 30% (*v/v*) complete Freund adjuvant (CFA) (F5881, Sigma-Aldrich, St. Louis, MO, USA) in saline, boosted two weeks later with 1.0 mg rRBD in 30% (*v/v*) incomplete Freund adjuvant (IFA) (F5506-Sigma-Aldrich) *v/v* in saline and boosted 3–4 times at weekly intervals with 1.5 mg rRBD in a 20% (*v/v*) dilution in saline of a stock Al(OH)_3_ suspension. Then, 60–120 days after the end of the I cycle, a second immunization cycle (R cycle) was performed. During R cycle horses were initially primed subcutaneously with 0.5 mg rRBD in 30% (*v/v*) incomplete Freund adjuvant (IFA) *v/v* in saline and boosted 3–4 times at weekly intervals with 1.5 mg rRBD 20% (*v/v*) dilution in saline of a stock Al(OH)_3_ suspension. Samples of hyperimmunized horse’s serum (HHS) were collected at each immunization and stored at −80 °C until use. Seven days after receiving their last immunization, during each I or R cycles, horses’ blood was extracted in two sequential days, their plasma obtained by citrate addition, separated and conserved refrigerated until use for Anti-SARS-CoV-2 production.

Indirect ELISA: In total, 150 µL HHS samples, diluted 1:4000 in PBS-2% (*w*/*v*) skim milk, were added to the wells of Maxisorp^®^ (Thermo Fisher Scientific, Waltham, MA, USA) 96 wells ELISA plates, previously coated with 100 ng rRBD per well and incubated for 60 min at 37 °C. The wells were then washed and incubated with a 1:50,000 dilution of a peroxidase-labeled anti-horse IgG (A6917, Sigma-Aldrich, St. Louis, MO, USA) for 60 min at 37 °C. After discarding the secondary antibody, the wells were washed, incubated with TMB (3,3′,5,5′-tetramethylbenzidine) and color development was stopped by adding 2 M sulfuric acid. Color development was quantified by 450 nm optical density reading (OD_450 nm_) in an ELISA plate reader (Tecan GmbH, Grödig, Salzburg, Austria).

Avidity ELISA: Avidity in HHS samples was estimated as described by Björkmann et al. [20]. Avidity was defined as the quotient between urea-treated wells and urea-non treated wells of duplicates from each HHS sample (expressed as percentage).

Viral Infection Neutralizing Titer: To allow for straight comparison with most current reports from literature, we performed the most frequently described microneutralization assay version using live virus [21]. Briefly, Vero C1008 (clone E6, ATCC^®^ CRL-1586™) were cultured in DMEM (Sigma-Aldrich) supplemented with 5% heat-inactivated fetal bovine serum (FBS, Sigma-Aldrich, St. Louis, MO, USA), 2 mM L-Glutamine (Sigma-Aldrich, St. Louis, MO, USA), penicillin (100 U/mL, Sigma-Aldrich, St. Louis, MO, USA), and streptomycin (100 µg/mL, Sigma-Aldrich, St. Louis, MO, USA). Serum samples were heat-inactivated (56 °C for 30 min) and serial dilutions (1/160 to 1/163840) were incubated (37 °C for 1 h) in the presence of wild type SARS-CoV-2 (moi = 0.01) in DMEM 2% FBS (hCoV-19/Argentina/PAIS-G0001/2020, GISAID Accession ID: EPI_ISL_499083; provided by Sandra Gallego InViV working group). Then, 50 µL of the mixtures were deposited over Vero cells monolayers for 1 h at 37 °C. Infectious media was removed and replaced by DMEM 2% FBS. After 72 h, cells were fixed with PFA 4% (*w*/*v*) (20 min at 4 °C) and stained with crystal violet solution in methanol. Neutralization titer (NtAb) was defined as the highest serum dilution without any cytopathic effect on the cell monolayer in two of three replicated wells.

Statistical Analysis: Normal distribution was assessed by Shapiro–Wilk test of normality. Contrast analysis was performed by the Welch’s version of Student’s *t*-test. Correlation between pairs of experimental groups of observations was estimated by Spearman’s rho factor. The influence of time on anti-rRBD IgG antibodies in horses’ sera (estimated as OD_450 nm_ of indirect ELISA experiments) was fitted to nonlinear models. Population-based, single individual and subject-specific fitting of experimental data to nonlinear models was undertaken using nls, nlsList and nlme functions, respectively, from nlme package of R software [22,23]. Experimental data was grouped after the cycle (I or R) from which their sera were obtained. Detailed information can be obtained from the Supplementary Material. Graphical representation of experimental data was performed using ggplot2 package of R software [23,24].

## 3. Results

### 3.1. Higher RBD-Specific Antibody Levels and Viral Neutralizing Titers Are Induced in Horses’ Blood during Shorter Hyperimmunization Cycles in Previously Hyperimmunized Animals

**Figure 1:**
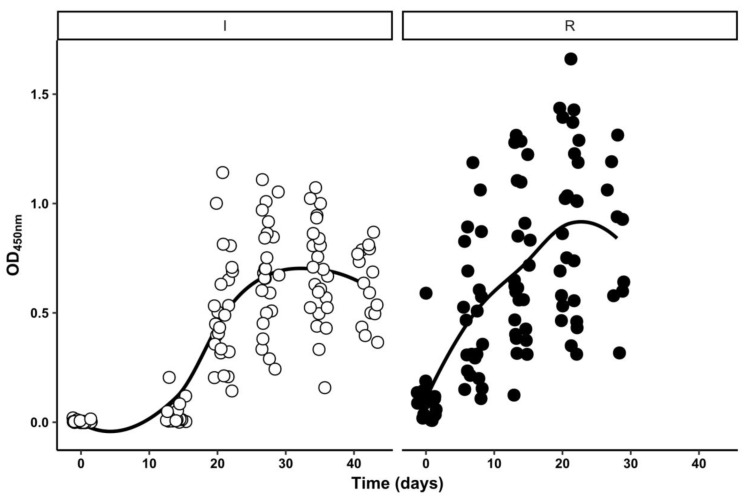
rRBD-specific IgG immune response kinetics in horses’ sera. Blood was extracted from the jugular vein of horses at regular intervals during each immunization cycle. RBD-specific IgG response in sera obtained from these blood samples was estimated by the indirect ELISA technique described in Section 2. Figure 1 displays the level of rRBD-specific IgG response detected (OD_450 nm_) for each sera sample at the different times of extraction (Time). Results of sera obtained during the initial immunization cycle (white circles) and during the following reimmunization cycle (black circles) are represented together with their estimated group means (black lines).

Hyperimmune horses’ sera (HHS) were obtained from blood extracted at regular intervals during both SARS-CoV-2 rRBD immunization (I) and reimmunization (R) cycles, as described in Section 2. The immune response kinetics in HHS from I and R cycles was characterized by a home-made indirect ELISA, which uses rRBD as the capture antigen (Figure 1).

Figure 1 shows that while anti-rRBD IgG antibodies response starts almost immediately at the beginning of the R cycle in HHS, there is an approximately two-week lag before this increase is observed in the I cycle. Additionally, anti-rRBD IgG antibodies levels seem to reach a plateau at a lower level during the I cycle when compared to that of the R cycle. Data analysis was performed by fitting different nonlinear models to the experimental data (Figure S2). Starting from simple population-averaged models (Figure S3, Table S1, Figure S4 and Table S2), then parameter intensive single-individual models were built (Figure S5) that ultimately helped to construct more parsimonious mixed-effects models (Figures S6 and S7). Analysis of the estimated parameters of these final nonlinear mixed-effects models (Table S3) led us to conclude that half-maximal OD_450 nm_ levels were reached at shorter times during the R cycle (7.0 days vs. 18.7 days). OD_450 nm_ were also higher at the end of the R cycle (0.87 vs. 0.67). The observed differences in anti-rRBD IgG responses among horses from both groups were related mostly to the different potential of each horse to reach higher OD_450 nm_ at the end of each cycle rather than to other kinetical parameters, i.e., initial OD_450 nm_ levels, steepness of the response or time to achieve half-maximal levels.

Viral neutralization potential (NtAb) is the critical parameter that qualifies HHP aptitude as starting material for immunotherapeutic production. The NtAb in HHS obtained from each horse seven days after receiving their final immunization dose was quantified by an ex vivo test using Vero cell line monolayers as the substrate. As described in Section 2, the microneutralization assay methodology was chosen for allowing a straight comparison with results of most current reports performed on sera of convalescent patients or vaccinated individuals where neutralization potential of live virus infection was evaluated.

**Figure 2:**
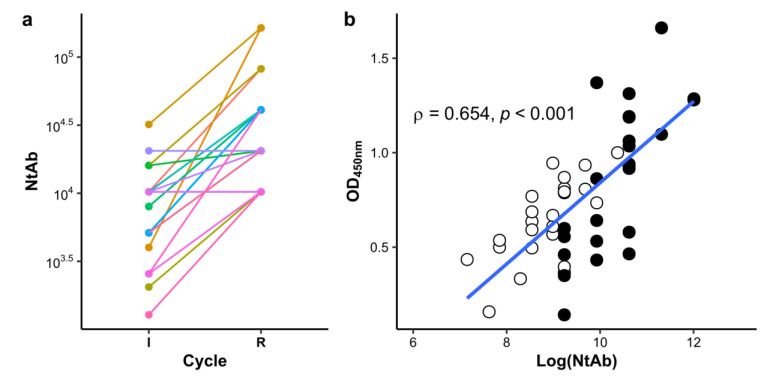
(**a**) NtAb contrast in HHS at final blood collection date during I and R cycles. HHS were collected from horses 7 days after receiving their last immunization dose. These HHS are the starting material for EpAbs production. Their viral infection neutralization titer (NtAb) was estimated as described in Section 2 and grouped according to their corresponding immunization cycles (as stated in *x*-axis). Paired dot and lines are represented for each horse to aid visual inspection and analysis of both individual and group difference. Statistical inference analysis between groups is included in the text. (**b**) Correlation analysis between indirect ELISA and viral neutralization results. Indirect ELISA results (OD_450 nm_) on HHS of horses from the I (white circles) and R (black circles) were plotted against log transformed viral neutralization antibodies titers (Log(NtAb)). Spearman’s rank correlation coefficient is depicted in the chart together with the *p* value associated with rejecting the null hypothesis (that OD_450 nm_ and Log(NtAb) are not correlated) whether it is true. A linear regression line is shown in blue to aid visual inspection of the data.

Shapiro–Wilk test of normality indicated that log normal transformation of crude NtAb values was necessary for achieving normal distribution in this variable (*p* < 0.05). As Figure 2 shows, higher NtAb were observed in HHS at the end of the R cycle (mean = 31,585 NtAb units) when compared to the I cycle (mean = 7000 NtAb units) (Welch’s *t*-test, *p* < 0.001). These results are not only consistent with those of Figure 1, but indirect ELISA OD_450 nm_ and NtAb results show significant correlation (Figure 2b, Spearman rho = 0.654, *p* < 0.001). Thus, ELISA results could be valuable estimators of NtAb, simplifying and thus lowering the bio risk associated to NtAb determination during quality control.

### 3.2. Increase in HHS NtAb Titers after a Second Hyperimmunization Cycle Correlates to Significant Antibodies Affinity Maturation

**Figure 3:**
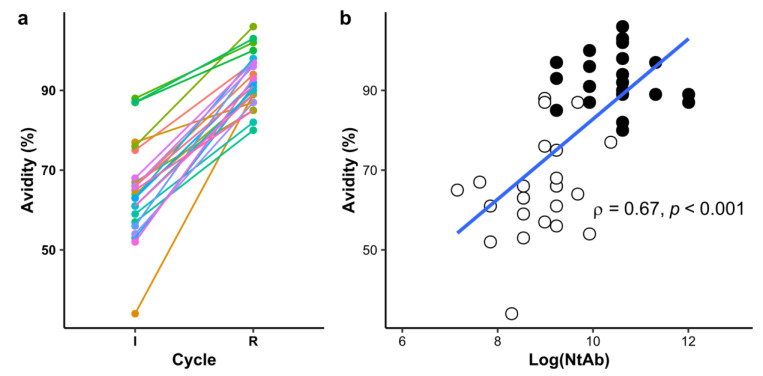
(**a**) Affinity maturation between hyperimmunization cycles. rRBD-specific avidity testing for IgG antibodies in HHS samples collected in each cycle at the moment of final blood extraction was analyzed as described in Section 2. Individual avidity scores (%) are grouped according to their respective immunization cycle (as stated in *x*-axis). Paired dot and lines are represented for everyone to aid visual inspection and analysis of both individual and group differences. Statistical inference analysis between groups is included in the text. (**b**) Correlation analysis affinity maturation and viral neutralization. Avidity on HHS of horses from the I (white circles) and R (black circles) were plotted against log transformed viral neutralization antibodies titers (Log(NtAb)). Spearman’s rank correlation coefficient is depicted in the chart together with the *p* value associated with rejecting the null hypothesis (that Avidity and Log(NtAb) are not correlated) whether it is true. A linear regression line is shown in blue to aid visual inspection of the data.

While at the beginning of the I cycle, antibody class-switching takes place, at later stages of the I cycle as well as during the R cycle, plasmatic cells multiplication, memory cell differentiation and paratope affinity maturation occur. Indirect ELISA OD_450 nm_ and NtAb titers reflect the combined effect of both avidity and concentration of specific antibodies in sera or plasma. To gain further knowledge on the quality of the polyclonal antibodies being produced, we estimated and compared the avidity of HHS samples at the end of I and R cycles by making slight changes to the original indirect ELISA (see Section 2 for further details). As is clearly shown in Figure 3, a significant increase in avidity is observed at the end of R with respect to I cycle (Welch’s *t*-test contrast between mean avidity in the groups, *p* < 0.001).

These results highlight the significant degree of avidity maturation that takes place during the R cycle. Noteworthy, even though three HHS showed similar NtAb at the end of both cycles, their avidity for rRBD was increased after the R cycle. Furthermore, avidity results were strongly correlated to NtAb results, thus suggesting that affinity maturation has a positive effect on viral neutralization (Figure 3b, Spearman rho = 0.670, *p* < 0.001).

## 4. Discussion

Initial reports on the effects of immunotherapeutic products based on neutralizing polyclonal antibodies or their fragments for treating COVID-19 patients at different stages of the disease are promising [4,8]. They could be valuable tools to help stressed public health in the struggle against the current COVID-19 pandemic in a context where mass vaccination is still lacking. The inherent complexity of these therapeutic agents, due to their polyclonal nature, should be considered. Most work in the field of anti-COVID-19 immunotherapeutics based on HHP antibodies or their fragments has been focused on developing high NtAb titers in naïve animals [10,11,12,13,14]. Additionally, these reports describe the effect of initial hyperimmunization cycles on limited groups of at most five animals. From a productive point of view, large animals used for HHP production are expected to receive several hyperimmunization cycles throughout their lives. Our work, analyzing both initial and reimmunization cycles on a group of 24 horses, allowed for thorough statistical analysis. Through nonlinear mixed-models we showed that the most important source of variation in both I and R cycles is related to the maximal achievable immune-response in each individual and not in the time elapsed to achieve this level. No beneficial effect was observed after administering more than four doses during both the I and R cycles. These two observations are most relevant for planning HHP production calendars and antigen requirements.

An important aspect when analyzing reports from different sources is that neutralization assays are not standardized. Thus, comparison between different reports usually remains elusive. However, in our group we performed the same neutralization assay to determine the neutralizing activity in HHS, vaccinated sera and convalescent sera in parallel. This intra-laboratory comparison revealed that horse hyperimmune sera present high neutralizing activity. In a recent report, we show that Sputnik V vaccine recipients had a geometric mean in their neutralizing antibody titer of 28 in naïve individuals and 782 for participants with prior SARS-CoV-2 infection. In marked contrast, HHS after the I cycle had a geometric mean of 7000 (CI95% 5096–9614; *n* = 24) and after the R cycle of 31,585 (CI95% 22409–44519; *n* = 24) [25]. This highlights the level of NtAb achieved in HHS and supports the potential of HHP for anti-SARS-CoV-2 EpAbs production.

Furthermore, as we have described across our work, significant affinity maturation occurs during repeated hyperimmunization cycles. NtAb test, perhaps the most relevant efficacy-related quality control test currently used, is an oversimplification of the viral neutralization that takes place when these products are administered by the parenteral route. In the complex in vivo scenario, avidity might play an important role that is potentially under-represented during NtAb testing. Recent studies suggest that avidity maturation during infection might have a significant effect on the prognosis of the disease [26]. By selecting those HHP with higher avidity, better quality in the final product would be expected. Thus, we propose that avidity testing, a low-cost but seemingly relevant technique, should be considered for process control routines during production of these complex polyclonal immunotherapeutic agents.

## Data Availability

All the data from this study is available through contact to correspondence author.

## Supplementary Materials

The following are available online at https://www.mdpi.com/article/10.3390/antib11010003/s1, Supplementary Material: Antigen characterisation by sodium dodecyl sulphate denaturant polyacrylamide gel electrophoresis (SDS-PAGE): Figure S1: SDS-PAGE characterization of a representative rRBD lot. Statistical Modelling of Specific Anti-RBD IgG Response: Figure S2: Representation of experimental anti-RBD specific IgG response during the immunization (panel A) and reimmunization (panel B) periods; Figure S3: Nonlinear modelling of the average population dependency between OD450nm and time for data from the immunization (panel A) and reimmunization (panel B) periods; Figure S4: Standardized residuals plots for fitted nonlinear models for horses from the I and R periods (panel A and B, respectively); Figure S5: Estimated parameters of individual nonlinear models for the IgG anti-RBD specific immune response in sera of horses from I and R periods (panel A and B, respectively); Figure S6: Residuals plot analysis of nonlinear models; Figure S7: Model fitting and experimental data; Table S1: Statistical population-based model formulae for data from each immunization period; Table S2: Fitting results of nonlinear least square estimation of model parameters; Table S3: Fitting results of nonlinear least square estimation of nonlinear mixed-effects models parameters.
