# Supplementary material for: Immune Maturation Effects on Viral Neutralization and Avidity of Hyperimmunized Equine Anti-SARS-CoV-2 Sera
Source: Antibodies (Basel). 2022 Jan 2;11(1):3. doi: 10.3390/antib11010003 (PMC8788445; doi:10.3390/antib11010003)
Supplement: Supplementary file 1 [file antibodies-11-00003-s001.zip › antibodies-1320381-supplementary.pdf]

Supplementary Material:

Antigen characterisation by sodium dodecyl sulphate denaturant polyacrylamide gel electrophoresis (SDS-PAGE):

Each lot of antigen (rRBD) was characterised prior to formulation. Antigen quality was assessed by SDS-PAGE under reducing and non-reducing conditions in 12,5 % p/v acrylamide and colloidal Coomassie brilliant blue stained. In the following figure an image of a representative rRBD lot (rRBD lot 0806) quality assessment is shown:

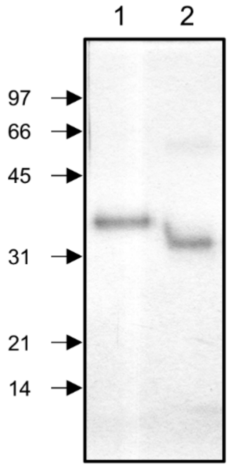

Figure S1: SDS-PAGE characterisation of a representative rRBD lot. SDS-PAGE resolution profiles of a sample of rRBD lot 0806 under reducing (lane 1) and non-reducing (lane 2) conditions. The gels were stained with colloidal Coomassie G250 Brilliant Blue using the standard methods, the images were digitalized with an office-scanner and analysed using ImageJ software (Version 1.51). Molecular weight references (in kDa) are indicated to the left of the image.

Statistical Modelling of Specific Anti-RBD IgG Response:

In this work anti-RBD IgG response evolution during hyperimmunization schedules was registered in terms of OD_{450nm} data from indirect ELISA experiments performed on sera samples collected at different time intervals. It is thus implicit the OD_{450nm} is expected to vary during the course of the experiment as a function of time. We could then try to formalize this relationship in terms of a longitudinal model where the response (OD_{450nm}) is assumed to vary according to a single covariate (time), following a previously defined function characterized by a set of parameters that are to be estimated based on maximum-likelihood criteria. Sources of random variation that can't be explained by this model (such as measurement inaccuracy) are included in an error term. Data modelling not only allows to extract statistical inference for hypothesis contrasts but its parameters also reflect relevant information about the general hypothesis (as will be shown later).

The data for each group of 24 horses (I and R groups) was analysed separately, beginning by visual inspection of experimental OD_{450nm} vs time graphical representation (Figure S1):

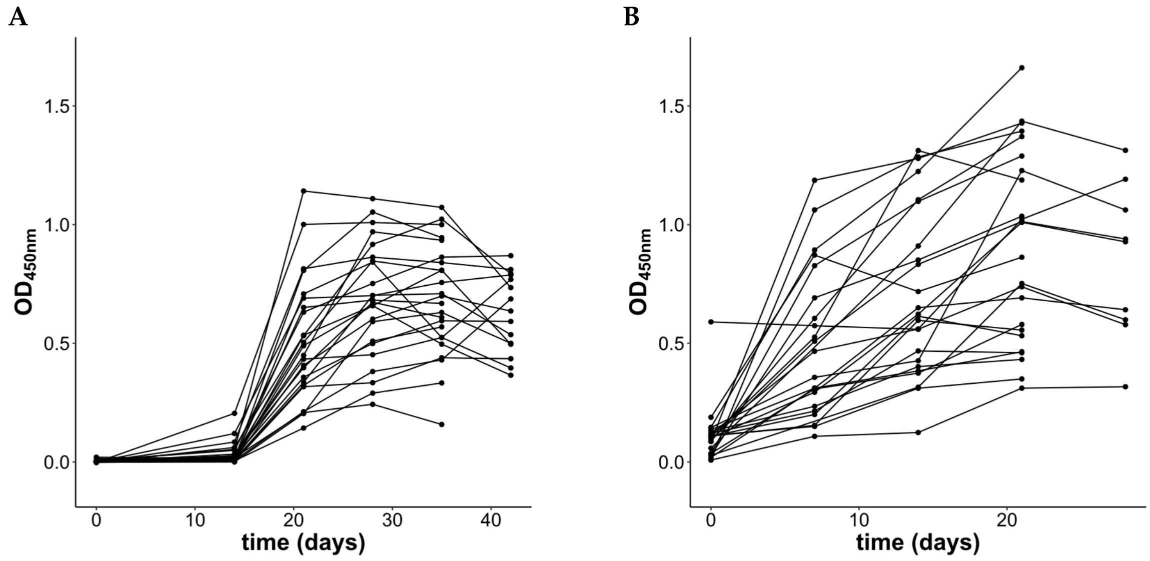

Figure S2: Representation of experimental anti-RBD specific IgG response during the immunization (panel A) and reimmunization (panel B) periods. The data obtained from sera of each different horse are represented with different colours.

It is clear from Figure S1 that the OD_{450nm} doesn't follow a linear tendency in time during any of the immunization schedules. Thus, nonlinear models were considered for modelling these data. In our initial approach, we constructed population-averaged models for both groups. These models consider these 24 horses as a randomly selected representative sample from a much larger population of horses and thus are intended to address questions that focus on the expected behaviour of the overall population. Although the number of individuals should be considered too small as to allow to extrapolate valid conclusions to the overall population, the population-averaged model are useful tools for an initial assessment of adequate functional models for posterior development of individual-specific models.

Let f (ϕ, t) be a function that models the dependency of the specific anti-RBD IgG response, (estimated through OD_{450nm}), in time, with its associated parameters described in the vector ϕ:

$OD_{450nm\ ij} = f(\phi, t_{ij}) + \varepsilon_{ij}$

where the residuals between the registered OD450nm for each individual horse i= 1,…, 24 at each time j=0,…, k days are assumed to have an independent, homoscedastic, normal random distribution modelled through the within-error matrix $\varepsilon_{ij} \sim N(0, \sigma^2)$. According with visual inspection of Figure S1, it was considered that while a four-parameter logistic model was adequate to model the experimental data from the I period, and simpler three-parameter logistic model was able to model the data from the R period (Table S1).

Table S1: Statistical population-based model formulae for data from each immunization period

**Period**	**Model formula**	**Parameters**
Immunization	$OD_{450nm} \sim f(\phi\ t_{ij}) = \phi 2 + \phi 1 / (1 + \exp[(\phi 3 - t_{ij}) / \phi 4]$	ϕ1 = <ODmax>, horizontal asymptote t → +∞<br>ϕ2 = (<ODmin>), horizontal asymptote t → -∞<br>ϕ3 = <OD50>, inflection point<br>ϕ4 = <scale>, scale parameter of the model.
Reimmunization	$OD_{450nm} \sim f(\phi\ t_{ij}) = \phi 1 / (1 + \exp[(\phi 2 - t_{ij}) / \phi 3]$	ϕ1 = <ODmax>, horizontal asymptote t → +∞<br>ϕ2 = <OD50>, inflection point<br>ϕ3 = <scale>, scale parameter of the model

Then, model fitting was performed by nonlinear (weighted) least squares estimation of the models parameters using the nls function of the nlme package from the open source statistical software R (Pinheiro and Bates, 2000). Results are represented in Figure S2 and the estimated parameters are described in Table S2:

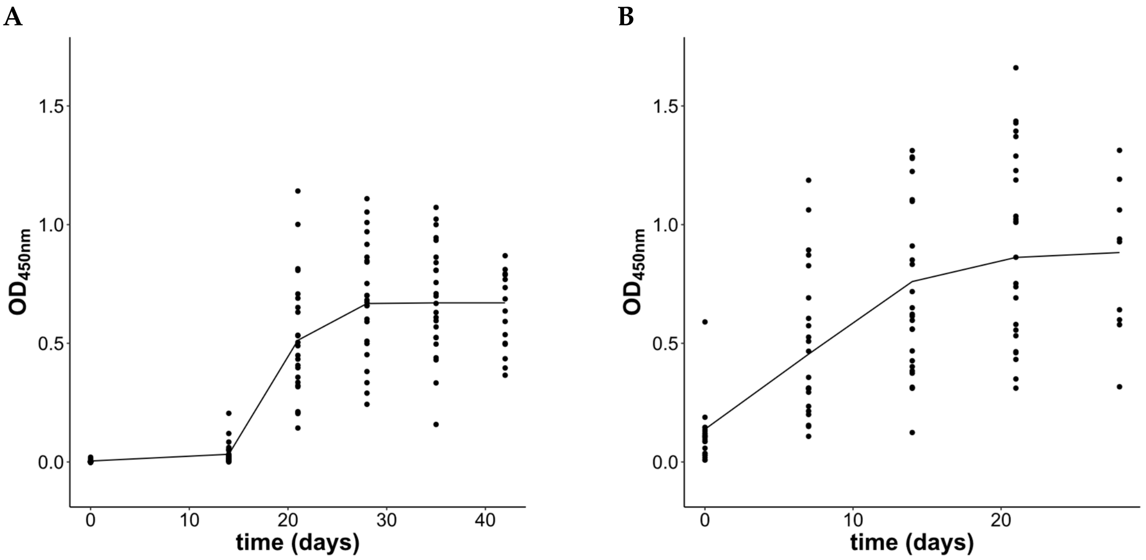

Figure S3: Nonlinear modelling of the average population dependency between OD_{450nm} and time for data from the immunization (panel A) and reimmunization (panel B) periods. The experimental data is represented by dots and the fitted model by a line.

Table S2: Fitting results of nonlinear least square estimation of model parameters

	**Nonlinear model**	
**Parameter** [CI $_{\alpha=0.95}$]	**Immunization**	**Reimmunization**
<ODmax>	0.67 OD unit [0.62 - 0.72]	0.89 OD unit [0.75 – 1.02]
<OD50>	19.1 day [17.1 – 21.1]	6.8 day [4.2 – 9.4]
<scale>	1.63 day [0.19 – 3.1]	4.0 day [1.7 – 6.3]
<ODmin>	0.004 OD unit [-0.07 – 0.08]	-
Within error (σ)	0.186 OD unit	0.316 OD unit

As already mentioned, a relevant aspect from nonlinear model fitting of experimental data is that it allows to extract conclusions from the estimated parameters of the model. In this case the <ODmax>, an estimation of the asymptotic or maximal OD_{450nm} value that is estimated to be reached at long time values, suggests that higher maximal OD_{450nm} values are expected during the R period when compared to the I period (0.89 vs 0.67, respectively). Moreover, <OD50>, the estimated time elapsed until the OD450nm reaches half-maximal levels, indicates that the immune response increases significantly faster during the R period when compared to the I period (19 days vs 7 days, respectively).

Noteworthy, these preliminary models do not take into account individual horses' contribution to variability, they are restricted to a population-averaged description. In this sense, as the residual plots in Figure S3 suggest, the within-error terms in both models are strongly influenced by systematic variability in individual horses' response to RBD immunization:

A

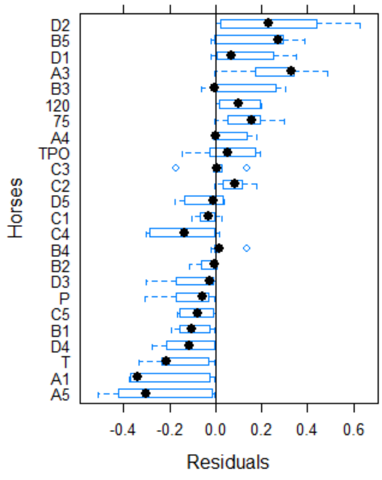

B

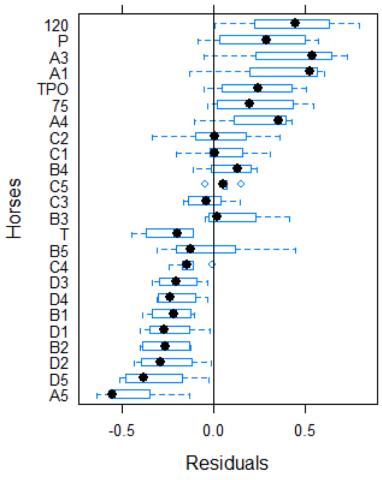

Figure S4: Standardized residuals plots for fitted nonlinear models for horses from the I and R periods (panel A and B, respectively). Mean values are represented as solid dots and standard error and confidence intervals are represented as boxes and whiskers, respectively.

A completely opposite approach for statistical modelling of experimental data would be to fit single nonlinear models for each individual horse from I and R groups. Nevertheless, since a full set of parameters must be fitted for each horse, this stresses the requisite for experimental data to follow the functional dependency of the model, thus limiting the possibilities of success during parameter estimation. Also, the extractable information from the parameters of this type of models is restricted to each horse, i.e., the individually fitted parameters are only valid for each and every horse and they do not represent general overall horses' response to RBD immunization. The model now takes the form:

$OD_{ij} = f(\phi_i, t_{ij}) + \varepsilon_{ij}$

Here, the functional part of the models are expressed similarly as in the population-averaged models constructed before, but in this case there is a ϕ parameters vector for each i horse belonging to R and I groups of data. Individual nonlinear models were then fitted using the nlsList function from the nlme package from R software.

Individual nonlinear models could be fitted for the IgG anti-RBD specific immune response of 16 and 18 horses of the I and R groups, respectively. The estimated parameters in these individual models, represented in Figure S4, allow to drive relevant conclusions, as will be later described.

A

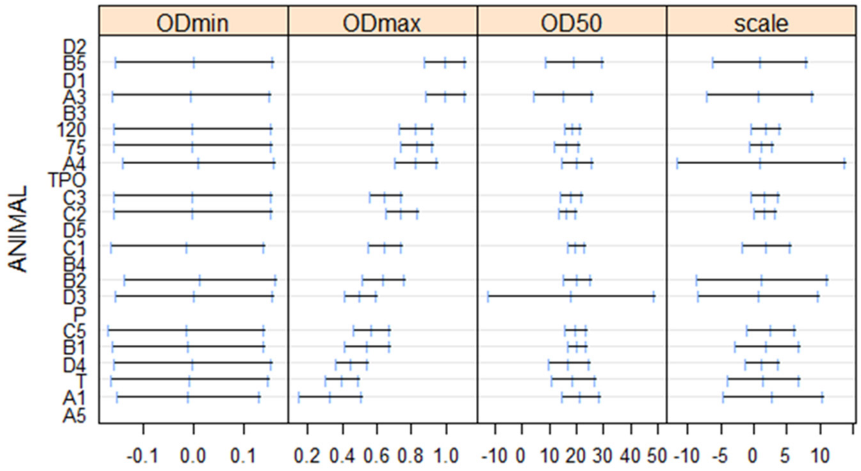

B

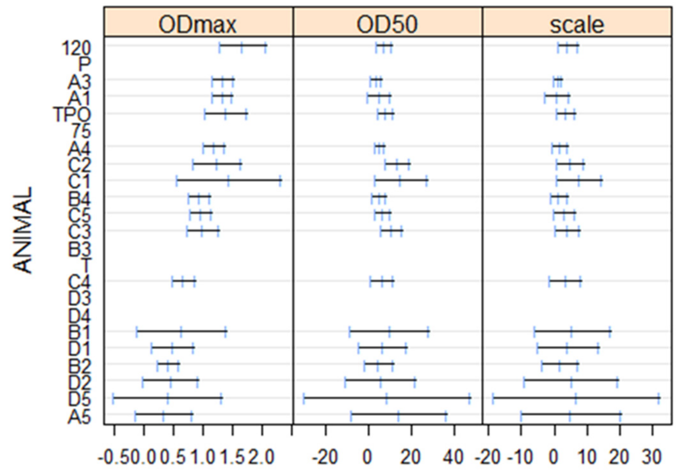

Figure S5: Estimated parameters of individual nonlinear models for the IgG anti-RBD specific immune response in sera of horses from I and R periods (panel A and B, respectively). Estimated values for each individual horse are represented with their corresponding confidence intervals.

As Figure S4 suggests, most parameters of nonlinear models for I and R groups overlap, with the exception of <ODmax>. This is indeed a relevant aspect of fitting single nonlinear models; these models show clues about how to model the random effects structure for nonlinear mixed-effects model building. We could thus build nonlinear mixed-effects models from the experimental data of each group of horses (I and R), where the dependency of OD450 with the covariate time follow the same functional dependency as in the latter models (three or four parameter logistic models for R and I group, respectively):

$OD_{ij} = f(\phi_{ij}, t_{ij}) + \varepsilon_{ij}$

Where $f(\phi_{ij}, t_{ij})$ for I and R groups share the same structure as described in table S1. But opposite to the initial population-averaged models in these subject-specific models, the parameters have an additive structure composed of two parts:

$\phi_{ij} = A_{ij}\beta i + B_{ij} b_i$, $\varepsilon_{ij} \sim N(0, \sigma^2)$., $b_i \sim N(0, \psi^2)$.

The first term in the formula models the information common to all the individuals in each group through the vector of fixed effects βi. The second term models the differences observed among individuals through the random independent normally distributed effects bi. Figure S4 suggests that mixed effects-models could be constructed for both group of horses in which the only parameter that should include random effects to account for differences among individuals is <ODmax>. The nlme package of statistical software R allows to estimate nonlinear mixed models, where the required initial estimates for the parameters can be extracted from the individual nonlinear models developed before. The estimated parameters after model fitting are shown in Table S3:

Table S3: Fitting results of nonlinear least square estimation of nonlinear mixed-effects models parameters

		**Nonlinear model**	
	**Parameter**	**Immunization**	**Reimmunization**
Fixed-effects	<ODmax>	0.67 OD unit [0.58-0.76]	0.87 OD unit [0.72-1.03]
	<OD50>	18.7 day [17.8-19.6]	7.0 day [5.4-8.6]
	<scale>	1.68 day [1.16-2.21]	2.8 day [2.2-3.4]
	<ODmin>	-0.003 OD unit [-0.04-0.03]	-
Random-effects	Within-error (σ)	0.09 OD unit [0.08-0.10]	0.12 OD unit [0.1-0.15]
	$SD_{<ODmax>}$ ∣ Horse	0.22 OD unit [0.16-0.29]	0.37 OD unit [0.27-0.5]
	$SD_{<OD50>}$ ∣ Horse	-	3.23 day [0.2-4.8]

The estimated fixed-effects of the parameters in these models closely resemble the estimated parameters from the initial population-averaged models. Again, higher <ODmax> are reached at shorter times (<OD50>) in the R group. Nevertheless, from a mathematical point of view it should be mentioned that although this part of the model represent the contribution of all the animals on each group, the nonlinear nature of model implies that the overall population mean of nonlinear mixed models is not identical to the initial population-averaged models.

The contribution from among-animals sources of variation is modeled through the random effects bi and the within-animals sources of variation is modeled through σ. As Table S3 shows, the estimated within-error is similar for both groups and significantly lower than estimated for population-averaged models. When the most important source of among-individuals variation from both groups, $SD_{<ODmax>}$ is normalized to its respective fixed effect ($SD_{<ODmax>}$/<ODmax>) it can be concluded that among-individuals variation seems to be higher for the R group when compared to the I group (0.43 vs 0.33, respectively).

Finally, the contribution of individual-specific models (the final mixed-effects models) to account for among-animals sources of variation is clearly seen when residual plots for the fitted models (Figure S5) are compared to those of population averaged models (Figure S3).

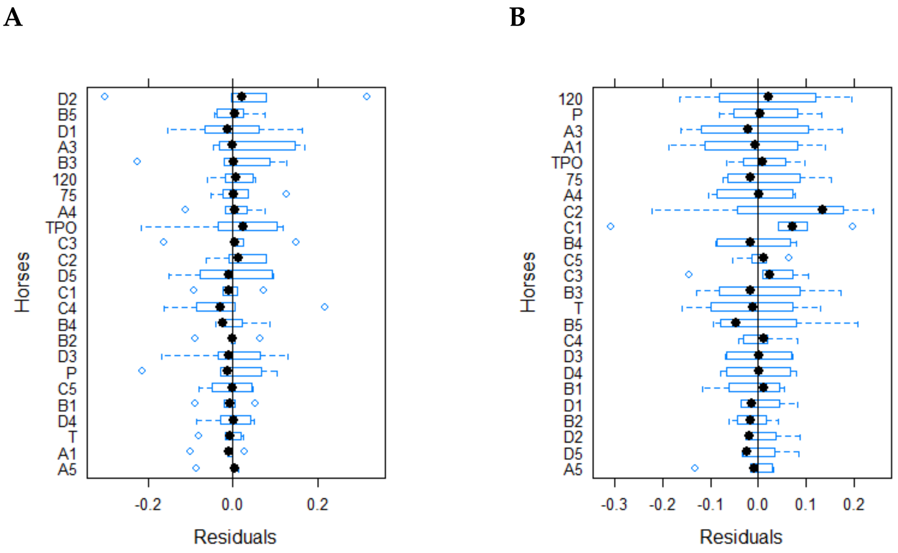

Figure S6: Residuals plot analysis of nonlinear models. Estimated residuals for each model were plot as crude raw values (displaying their median and standard errors, left panels) or as standardized values, against their fitted values (right panels).

Thus, a clear homogenizing effect for residuals variability (residuals = experimental OD_{450nm} minus fitted OD_{450nm}) between horses is observed through the introduction of mixed-models. NLME models are thus in closer agreement with assumptions made on within-error independency and homoscedasticity. Moreover, the closer fitting of NLME to experimental data for each individual horse is clearly shown in Figure S5:

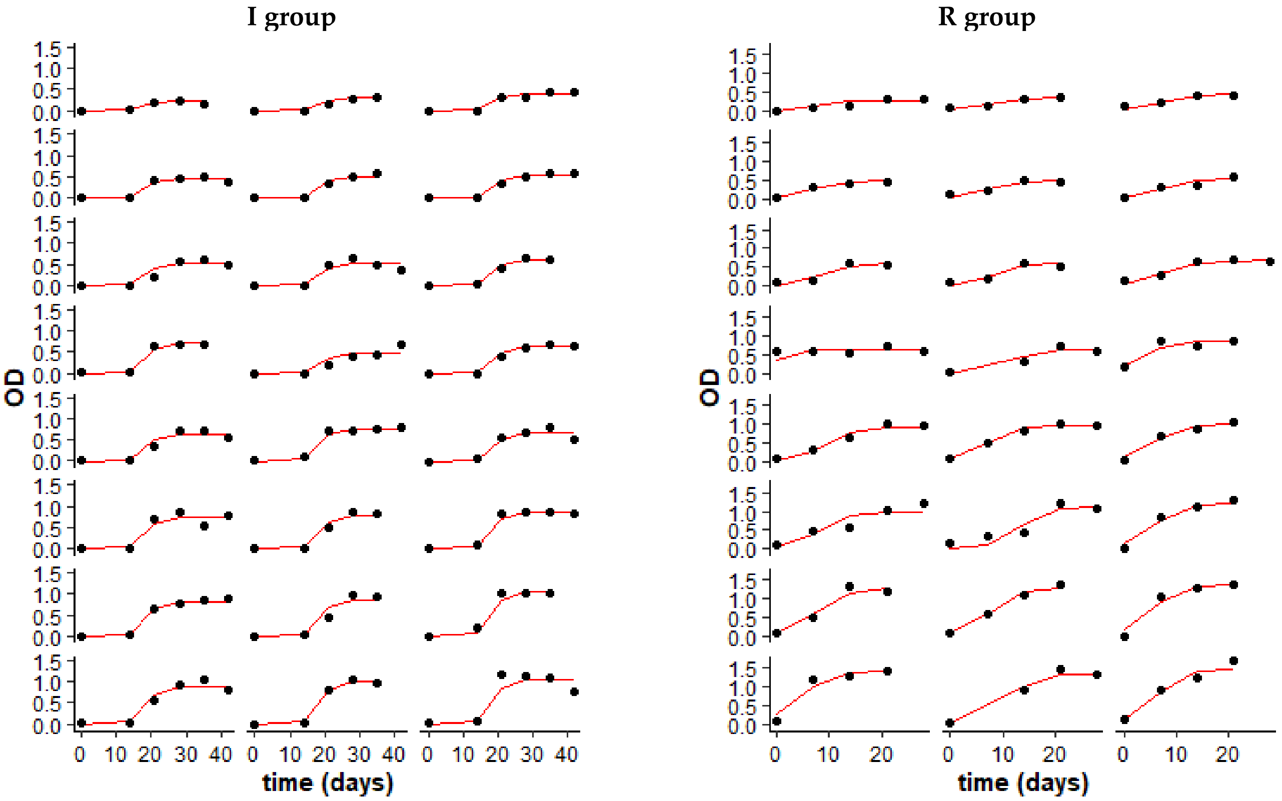

Figure S7: Mixed-effects model fitting to experimental data. Nlme models (red lines) are represented together with experimental data (black circles) for the I group (left panel) and R group (right panel).

Thus, as Figure S6 suggests, nonlinear mixed-effects models could adequately fit the experimental data obtained during RBD immunization. The differences in the estimated parameters for these models suggest that significantly higher final levels of immunization are obtained in shorter times during reimmunization. The random-effects components in these models, by accounting for among-individuals differences between horses inside each group, also suggest that for horses of both I and R groups the major source of variation between individuals is the maximal levels of specific IgG anti-RBD immune response achievable in horses' sera.
